# Neuronal avalanches and the cortico-striatal network

**DOI:** 10.1186/1471-2202-13-S1-P122

**Published:** 2012-07-16

**Authors:** Jovana J Belić, Andreas Klaus, Dietmar Plenz, Jeanette Hellgren Kotaleski

**Affiliations:** 1 Computational Biology, Royal Institute of Technology (KTH), Stockholm, 106 91, Sweden; 2 Bernstein Center Freiburg, University of Freiburg, Freiburg, 79104, Germany; 3 Department of Neuroscience, Karolinska Institutet (KI), Stockholm, 171 77, Sweden; 4 Section on Critical Brain Dynamics, National Institute of Mental Health (NIH), Bethesda, USA

## 

Neuronal avalanches are spontaneous activity cascades observed in superficial cortical layers with statistical properties expected from the network operating near a critical point [1]. In such a network, neuronal activity on one active site triggers, on average, similar activity at other site and therefore the overall activity does not increase or die out over time. Neuronal avalanches have been found *in vitro *[1] and *in vivo *[2], and display long-term stability, diversity, and fast propagation of local synchrony. They characterize networks that have a maximum dynamic range [3] and might play a central role in information transmission [1] and storage [4]. Their activity is characterized by brief bursts lasting tens of milliseconds, separated by periods of quiescence lasting several seconds and when observed with multi-electrode arrays, the number of electrodes activated is well described by a power law with exponent close to -1.5 [5].

Here we study neuronal avalanches in an open-loop system of cortex and striatum. The striatum is the main input structure of the basal ganglia and plays an important role in motor and cognitive functions. Understanding how the striatum responds to cortical inputs has crucial importance for clarifying the overall functions of the basal ganglia. The projection neurons of the striatum have a high threshold for activation and receive excitatory input from different regions of the cerebral cortex. Although the striatum contains several distinct cell types, 90-95% are GABAergic medium spiny projection neurons (MSNs). These cells are major targets of cortical inputs, the recurrent connection among them mediate weak feedback inhibition and neighboring MSNs are not likely to share cortical inputs [6]. Fast-spiking interneurons are relatively sparse elements of striatal networks. They project extensively to nearby MSNs and provide strong feedforward inhibition and seem to be critical nodes governing striatal output [7].

Preliminary experiments indicate that activity bursts in the striatum do not follow a power law with characteristic exponent of -1.5. Here we developed an abstract model of the cortico-striatal network that reproduces statistics observed in experimental data. We discuss which kind of connectivity between cortex and striatum, as well as connectivity and strength of inhibition within striatum can lead to results that are in line with experimental data.
